# Kinematics of transition during human accelerated sprinting

**DOI:** 10.1242/bio.20148284

**Published:** 2014-07-04

**Authors:** Ryu Nagahara, Takeo Matsubayashi, Akifumi Matsuo, Koji Zushi

**Affiliations:** 1 Faculty of Health and Sport Sciences, University of Tsukuba, Ibaraki 305-8574, Japan; 2 Japan Institute of Sports Sciences, Tokyo 115-0056, Japan; 3 National Institute of Fitness and Sports in Kanoya, Kagoshima 891-2311, Japan

**Keywords:** Biomechanics, Breakpoint, Locomotion, Running, Attractor

## Abstract

This study investigated kinematics of human accelerated sprinting through 50 m and examined whether there is transition and changes in acceleration strategies during the entire acceleration phase. Twelve male sprinters performed a 60-m sprint, during which step-to-step kinematics were captured using 60 infrared cameras. To detect the transition during the acceleration phase, the mean height of the whole-body centre of gravity (CG) during the support phase was adopted as a measure. Detection methods found two transitions during the entire acceleration phase of maximal sprinting, and the acceleration phase could thus be divided into initial, middle, and final sections. Discriminable kinematic changes were found when the sprinters crossed the detected first transition—the foot contacting the ground in front of the CG, the knee-joint starting to flex during the support phase, terminating an increase in step frequency—and second transition—the termination of changes in body postures and the start of a slight decrease in the intensity of hip-joint movements, thus validating the employed methods. In each acceleration section, different contributions of lower-extremity segments to increase in the CG forward velocity—thigh and shank for the initial section, thigh, shank, and foot for the middle section, shank and foot for the final section—were verified, establishing different acceleration strategies during the entire acceleration phase. In conclusion, there are presumably two transitions during human maximal accelerated sprinting that divide the entire acceleration phase into three sections, and different acceleration strategies represented by the contributions of the segments for running speed are employed.

## INTRODUCTION

Sprint running (sprinting) is a high-speed locomotion mode. The winner of the 100-m race at the highest competitive level is considered the fastest man or woman in the world at that time. The 100-m race time is strongly correlated with maximal sprinting speed during the race (Bruggemann and Glad, 1990; Maćkala, 2007; Volkov and Lapin, 1979), and the time during which the sprinter can accelerate with maximal effort is limited to 5–7 seconds (Hirvonen et al., 1987; Margaria et al., 1966). Because the maximal sprinting speed depends on the preceding increase in speed in the acceleration phase, the ability to accelerate is critical to 100-m race performance (Doolittle and Tellez, 1984; van Ingen Schenau et al., 1994).

There are two typical running modalities during the entire acceleration phase of maximal sprinting (Dillman, 1975). During the initial acceleration phase, especially at the first step, sprinters have a deeply hanging posture (i.e. a trunk leaning forward) that assists acceleration as the whole body's centre of gravity (CG) is brought ahead of the base of the support and positioned close to the ground reaction force (GRF) vector (Debaere et al., 2013b; Kugler and Janshen, 2010; Novacheck, 1998; Celik and Piazza, 2013; Plamondon and Roy, 1984; Young et al., 2001). In contrast, at maximal speed, sprinters adopt an upright posture, as they cannot exert a propelling force that cancels the downward gravitational moment (Bosch and Klomp, 2005; Kunz and Kaufmann, 1981; Novacheck, 1998; Plamondon and Roy, 1984; Young et al., 2001). There are also great changes in the spatiotemporal variables of sprinting from the first step to maximal speed phase. The step length (SL) increases (Bruggemann and Glad, 1990; Gajer et al., 1999; Maćkala, 2007; Nagahara et al., 2014; Plamondon and Roy, 1984), the support time (ST) decreases (Nagahara et al., 2014; Plamondon and Roy, 1984), and the flight time increases (Nagahara et al., 2014; Plamondon and Roy, 1984) with an increase in sprinting speed during the entire acceleration phase of maximal sprinting. Moreover, different manners of motions are recommended to sprint effectively; e.g. during the acceleration phase, speed development depends mainly on the powerful extensions of all major lower-extremity joints (Debaere et al., 2013b; Johnson and Buckley, 2001), and when the athlete reaches higher speeds, it is necessary to rotate the legs forwards and backwards relative to the hip joint and this limits a further increase in sprinting speed (van Ingen Schenau et al., 1994).

Owing to the aforementioned difference in running modalities that are adopted just after the start and at the maximal speed phase of sprinting, a concept of dividing the entire acceleration phase into sections has been developed (Bret et al., 2002; Debaere et al., 2013a; Delecluse, 1997; Maćkala, 2007; Nagahara et al., 2014). For instance, Nagahara et al. revealed that the entire acceleration phase of maximal sprinting can be divided into three sections, according to the changes in relationships between acceleration on the one hand and rates of changes in SL and step frequency (SF) on the other (Nagahara et al., 2014). Moreover, they supposed that the difference in the relationship of the acceleration with the rate of change in SL or SF responsible for the divisions is caused by changes in a pattern of running motion (Nagahara et al., 2014). On a practical basis, it has also been speculated that the running kinematics change abruptly between consecutive sections in what is called “transition” (Bosch and Klomp, 2005). However, it is still unknown whether there is a critical discrimination as a transition.

Although many studies have discussed various biomechanical aspects of sprinting, almost all are based on analyses at the specific spot of sprinting (Bezodis et al., 2008; Bezodis et al., 2014; Chapman and Caldwell, 1983; Charalambous et al., 2012; Debaere et al., 2013b; Hunter et al., 2004; Hunter et al., 2005; Jacobs and van Ingen Schenau, 1992; Johnson and Buckley, 2001; Kunz and Kaufmann, 1981; Mann and Herman, 1985; Mero et al., 1992; Nummela et al., 1994; Slawinski et al., 2010; Slawinski et al., 2013; Weyand et al., 2000). There are a small number of reports of what happens when the sprinter actually accelerates through multiple consecutive steps during the acceleration phase (Cavagna et al., 1971; Fukunaga et al., 1981; Morin et al., 2010; Morin et al., 2012; Nagahara et al., 2014; Plamondon and Roy, 1984), and there has never been detailed kinematic analysis of the entire acceleration phase of maximal sprinting. Knowledge gained from such conditions provides insights into the manner in which kinematics of a sprinter change through the discriminating transition point of the acceleration phase. The study of kinematics during the entire acceleration phase of maximal sprinting of human participants clarifies the function of the human bipedal locomotor system in the extreme condition.

In this study, kinematic data for the entire acceleration phase (from the first step to the 50-m mark) of human maximal sprinting were obtained to clarify the consecutive changes in kinematics of accelerated sprinting. The aims of this study were (1) to verify whether there is a transition during the maximal accelerated sprinting and (2) to demonstrate the change in acceleration strategy based on the kinematic measures during the entire acceleration phase of maximal sprinting.

## MATERIALS AND METHODS

### Participants

Table 1 gives the characteristics of 12 male Japanese sprinters who volunteered for this study. All participants were sprint (100 m and 200 m) specialists, and they were healthy and free from injury. The purposes, risks of involvement, and experimental conditions of the study were explained before the experiment, and written informed consent was obtained. The experimental procedures were conducted with the approval of the research ethics committee of the institute.

**Table 1.**
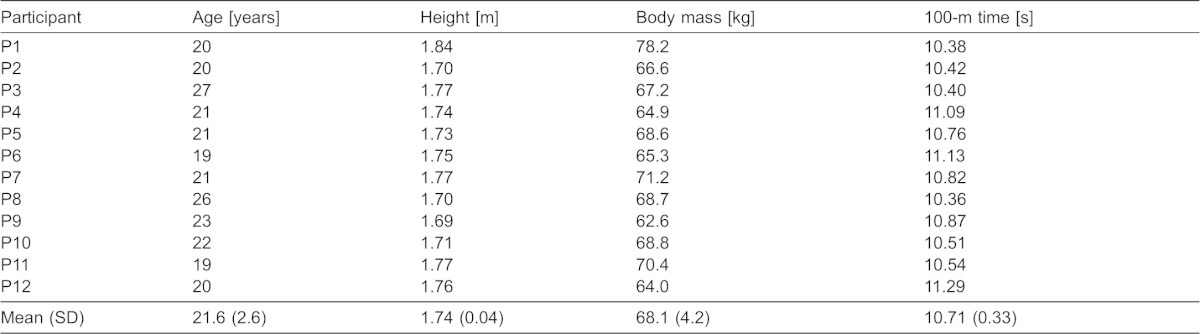
Characteristics of participants

### Experiments

After warming up, the participants twice performed a maximal-effort 60-m sprint. The sprint was treated as a 100-m race with starting blocks used and the participants using their own crouched starting position. Between trials, participants rested at least 15 minutes. All participants wore their own spiked shoes. The 60-m sprint time was measured using a photocell system connected to the starting gun (HL2-35, Tag Heuer, La Chaux-de-Fonds, Switzerland) and the start signal was recorded by a computer as a trigger signal.

**Fig. 1.**
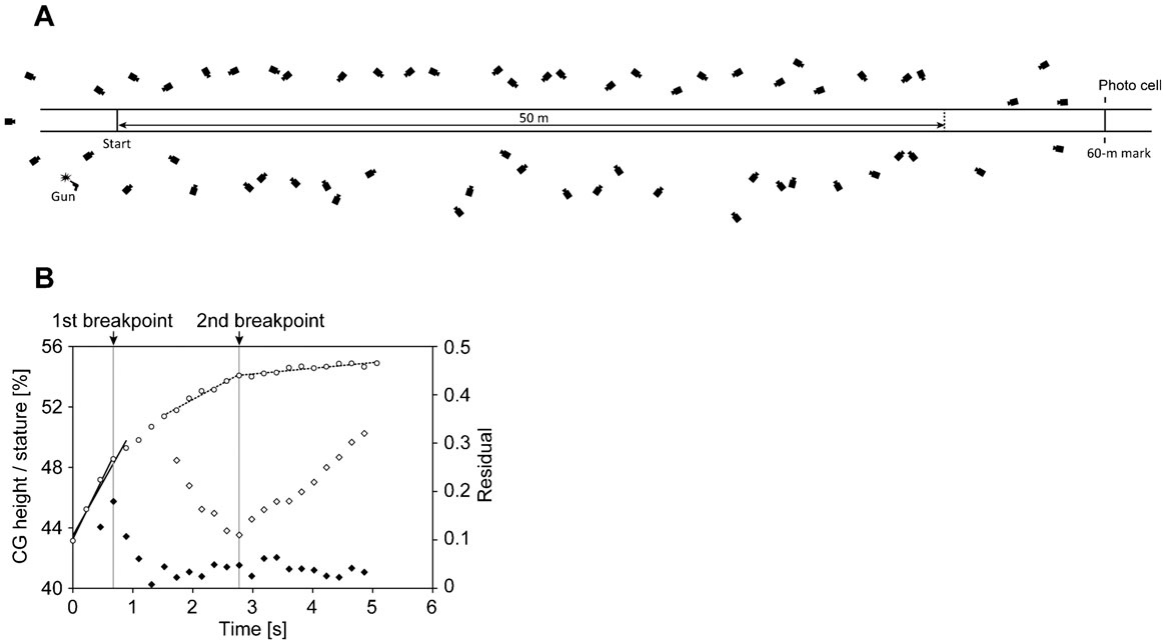
Experimental set-up and example of breakpoint detection. (A) Experimental set-up with 60 cameras. (B) Changes in average CGMH for the best trials of all participants and examples of detections of the first and the second breakpoints employing two methods. Open circles indicate the CGMH at each step. The data are plotted against average times from the first foot-strike to the foot-strikes of the respective steps. Solid lines at left are two approximation lines for four and five steps from the first step for detecting the first breakpoint. Dotted lines at middle and right are two approximation lines for detecting the second breakpoint. Closed diamonds are the differences in mean absolute residuals of two adjacent linear approximations. Open diamonds are the mean absolute residuals of the two linear approximations. Vertical solid grey lines indicate the two breakpoints.

Sixty infrared cameras (18 MX-T10, 30 MX-T20, and 12 MX-T40 cameras, 250 Hz) connected to a single computer through four MX Giganet devices (Vicon Motion Systems, Oxford, UK) captured three-dimensional coordinates of 47 retro-reflective markers affixed to the participant's body with a volume (length×width×height) of ∼50 m×1.5 m×2 m (Fig. 1A). The markers were placed on the third metacarpal heads of dorsal hands, styloid processes of ulnas and radii, medial and lateral epicondyles of humeruses, anterior and posterior parts of shoulders, tops of the acromions, toes, posterior of calcaneuses, medial and lateral parts of the first and fifth metatarsal heads, malleoli, femoral condyles, great trochanters, vertex, tragions, anterior and posterior of the suprasternal notch and xiphoid process, lateral lowest points of the ribs, anterior superior iliac spines, and posterior superior iliac spines. Segment end points were calculated from the three-dimensional coordinates of the markers according to a 15-segment body model consisting of hands, forearms, upper arms, feet, shanks, thighs, head, upper trunk, and lower trunk. End points were estimated depending on the joint or body segment in question. Markers affixed to the vertex, right and left of the third metacarpal heads of dorsal hands, toes, and posterior of calcaneuses were considered as end points of the segments. The midpoints of the markers affixed to the styloid processes of ulnas and radii, medial and lateral epicondyles of the humeruses, anterior and posterior parts of the shoulders, malleoli, and femoral condyles were taken as the joint centres of the wrists, elbows, shoulders, ankles, and knees, respectively. The midpoints of the markers affixed to the anterior and posterior parts of the suprasternal notch and left and right of the lateral lowest points of the ribs were respectively considered as the proximal end point of the head segment and the division point of the upper trunk and lower trunk. The hip joint centre was estimated using the method recommended by the Japan Clinical Gait Analysis Forum (A manual for the use of data interface file of gait analysis, 1992, Kanagawa (in Japanese)) as the most suitable for Japanese individuals, where the hip joint is defined as the point located 18% of the distance between the right and left great trochanters medially from the point located at one-third of the distance from the greater trochanter to the anterior superior iliac spine. The midpoint of the two hip joint centres was taken as the distal end point of the lower trunk. The end point coordinates were smoothed using a Butterworth digital filter at cut-off frequencies obtained using the residual method of Wells and Winter (Wells and Winter, 1980). The cut-off frequencies ranged from 17.5 to 22.5 Hz.

### Data processing

#### CG relative and spatiotemporal variables

After reconstructing the data to two dimensions in the sagittal plane, the position of the CG was calculated with the 15-segment body model, using body segment parameters of Japanese athletes that were obtained with a procedure similar to Jensen's mathematical modelling (Ae et al., 1992b; Jensen, 1978), and by adding the typical mass of a running shoe (200 g) (Hunter et al., 2004). Prior to the main experiment, we validated the kinematic-data-based estimation of the CG position (Eames et al., 1999; Segers et al., 2007). Five male sprinters (mean ± SD: age, 22.6±1.8 y; stature, 1.75±0.06 m; body mass, 65.5±4.7 kg; personal best 100-m time, 11.00±0.19 seconds) ran 5, 15, and 25 m three times with maximal effort from starting blocks. At the second, sixth, and tenth step during the individual sprints, the coordinate data of the sprinters were obtained with the same experimental procedures as used in the main experiment with 10 cameras (MX-T20), and the GRFs were recorded using a force plate (Kistler, Winterthur, Switzerland, model 9287C, 1000 Hz). CG positions of sprinters in all trials were estimated with the same body segments model in the main experiment. Additionally, CG positional displacements during the support phase were reliably calculated through the double integration of acceleration, which was deduced from GRFs, with respect to time. For 45 trials, CG displacements during the support phase calculated from the GRFs were compared with the associated CG displacements obtained from the kinematic data. Average measures of intra-class correlation coefficients were calculated and the values ranged from 0.90 to 0.98 for the vertical direction and were above 0.99 for the anterior–posterior direction (*P*<0.01). This indicates that the estimation of the CG position during accelerated sprinting using the kinematic data are reliable. Although we could not validate the estimation of the CG position with kinematic data for all steps during the entire acceleration phase as recorded in the main experiment, the results support the use of the kinematically estimated CG position and its change during maximal accelerated sprinting. The velocities of the CG were calculated by differentiating the kinematically estimated CG positions with respect to time.

To identify instants of the foot-strike and toe-off automatically, detection methods, using the instant of peak vertical acceleration of the toe marker for the foot-strike (Hreljac and Marshall, 2000; Nagahara and Zushi, 2013) and the next frame from the instant of the minimal vertical position of the toe marker for the toe-off (Nagahara and Zushi, 2013), were employed for all steps. The errors of these detection methods during the acceleration phase of maximal sprinting are plus or minus one frame when cameras are operating at 250 Hz (Nagahara and Zushi, 2013). Although the range of errors for the event detection is acceptable, the possible temporal error in the spatiotemporal variables was two frames (0.008 seconds) because it is possible that there were errors on both sides of a step or support phase. Therefore, it would be important to keep in mind the magnitude of the possible maximal errors as a limitation of the present study. SF was calculated as the inverse of step duration, which was determined from the foot-strike of one leg to the next foot-strike of the other leg. SL was calculated as the anterior–posterior distance between CG positions at the foot-strike of one leg and at the next foot-strike of the other leg. ST was obtained as the duration from the foot-strike to just before the toe-off. Support distance (SD) was the anterior–posterior distance that the CG moved during the support duration. Anterior support time (AST) was determined as the duration when the CG was behind the metatarsal phalangeal (MP) joint (centre of the markers on the medial and lateral parts of the first and fifth metatarsal heads), which was considered the location of the centre of pressure, of the support leg. The posterior support phase was determined as the duration when the CG was in front of the MP joint. Anterior support distance (ASD) was obtained as the anterior–posterior distance from the CG to the MP joint of the support leg at the foot-strike. The data from the first step after the starting blocks were cleared were used. Thus, the phase while feet were on the blocks was excluded.

#### Kinematics of segments and joints

The linear and angular kinematics of segments and joints were calculated. Angles of the segments were determined as the angles from the horizontal forward line to the segments comprising the vectors from the proximal ends to the distal ends (noting that the counter-clockwise direction from the right side view is positive). Joint angles of the hip and knee were the relative angles of the lower trunk and thigh (anterior side) and thigh and shank (posterior side). The direction of opening was considered as an extension of the joints. Angular velocities of segments and joints were calculated by differentiating the angles of the segments and joints with respect to time. The contributions of the lower-extremity segments to the CG horizontal velocity during the support phase, following the concept proposed in a previous study (Jacobs and van Ingen Schenau, 1992), were calculated by subtracting the anterior–posterior velocities of distal end points of segments from those of associated proximal end points for the thigh, shank, and foot (noting that, in the case of the foot, the distal end point was the ground). Although there remains a contribution from other components that can be calculated by subtracting the anterior–posterior velocity of the hip (proximal end point of the thigh) from the velocity of the CG, the contribution is expected to be very small and is thus ignored here.

#### Identification of breakpoints of the entire acceleration phase

To determine the breakpoint (transition step) that divides the entire acceleration phase into sections, a criterion (a variable associated with a detection method) was needed. The CG height was adopted as the criterion for breakpoint detection because the CG reflects the change in movements of the lower extremities and trunk, and the CG can be reliably measured according to the results of the aforementioned sub-experiment. Changes in the average of the mean CG height relative to the stature during the support phase (CGMH) for all participants, which are plotted against average time from the first step to each step in Fig. 1B, seemed to comprise three regions, as mentioned in previous studies (Debaere et al., 2013a; Delecluse, 1997; Nagahara et al., 2014), according to the gradients, and it was expected that two breakpoints would be determined during the entire acceleration phase.

To detect the first breakpoint, we adopted a modified method, using a straight-line approximation, which was used in a previous study determining ventilatory thresholds (Neder and Stein, 2006). When employing this method, we approximated the CGMH with a first-order equation with respect to time (where the duration of each step was from the foot-strike of the first step to the foot-strike of each subsequent step) for three steps at first, and the number of steps used in the approximation was then increased step to step toward the last step, while calculating the mean of the absolute residuals for each approximation. Afterward, differences in the mean absolute residuals between adjacent steps were calculated. The first variable of the difference in the mean absolute residuals was for the third step and the remaining variables corresponded to the fourth step to just before the last step. A maximal value of the difference in the mean absolute residuals was used as a criterion to detect the breakpoint, and the step where the criterion appeared was adopted as the first breakpoint step as shown in Fig. 1B.

Although the method used for the first breakpoint could not work for the second breakpoint, the second breakpoint was determined using the V-slope method with two straight-line approximations, which was developed to detect an anaerobic threshold (Schneider et al., 1993). This method is able to detect a breakpoint of continuous data that follow two rectilinear slopes joined at some unknown point (breakpoint) (Jones and Molitoris, 1984). When employing this method, the CGMH from the eighth to the last step was divided into two regions at first, and the point dividing the two regions was moved within the range of data while approximating with two first-order equations with respect to time for those regions and calculating the mean absolute residuals of both approximations. The inter-region step where the minimum value of the means of absolute residuals appeared was taken as the second breakpoint step (Fig. 1B). The method was applied only from the eighth step to eliminate the effect of the first breakpoint.

For both methods of detecting the transition step, the detection is considered incorrect if the magnitude of increase in CGMH becomes large across the transition step. Moreover, in regard to the method of detecting the first transition, when the detected transition step was beyond the seventh step, the detection was decided as an incorrect detection, because we expected that the first transition step is located from the third to seventh step owing to the profile of change in the average CGMH.

### Statistical analysis

Means and standard deviations of the variables at each step from the start and means and standard deviations of variables at each step before and after the breakpoints, which were calculated according to the individuals' breakpoint steps, were calculated. The variables for 25 steps were taken as mean variables at respective steps during the entire acceleration phase, because the smallest number of steps taken by a participant—the participant having the longest average SL—was 25. To test the difference in slope of changes in variables around the breakpoints, a simple two-tailed *t*-test was conducted. In the *t*-test, the following procedures were conducted in reference to a previous gait transition study (Segers et al., 2007). To eliminate the effect of a mediolateral difference and a variability in human cyclic movement, the values from two and four steps before the first and second breakpoint steps to the breakpoint steps and from the breakpoint steps to three and four steps after the first and second breakpoint steps were linearly approximated with respect to time. The coefficients of gradients before and after the respective breakpoint steps were then adopted as the variables for the *t*-test. The number of steps used in the *t*-test of the first breakpoint corresponded to the expectation that the changes in variables would be relatively acute and to the earliest breakpoint being at the third step, providing only two steps before the breakpoint, while changes in variables around the second breakpoint were expected to be relatively small. Although some variables do not change linearly during the entire acceleration, changes in variables in the aforementioned limited range of steps can be represented by linear lines with gradients that represent the magnitude of the changes in variables, as is done in this study. Statistical significance was set to 5%.

To illustrate the average changes in sprinting motion, the coordinates of the segment end points for the best trials of all individuals were standardised and averaged in accordance with a previously proposed method (Ae et al., 2007). To calculate the average motion, first, the time-series data of coordinates during the support phase were normalised. As a second step, the relative position from the CG to the coordinates of each segment end point was divided by the stature of the individual. Finally, the relative coordinates in relation to the CG for all individuals were averaged and multiplied by the average stature of all individuals.

## RESULTS

**Fig. 2.**
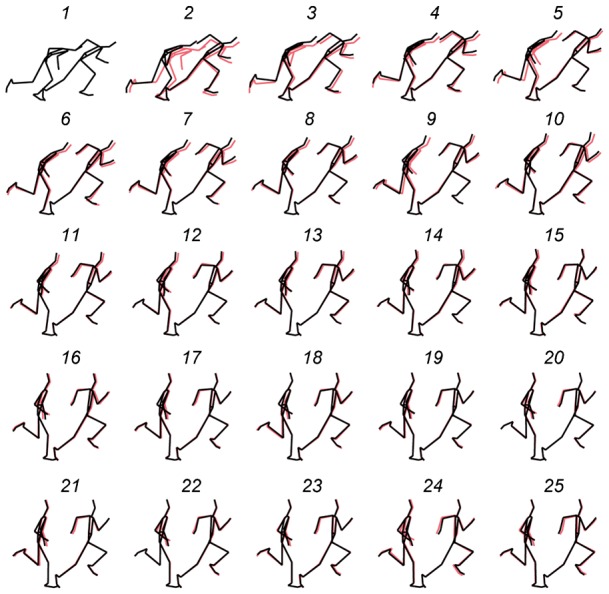
Changes in average sprinting motion during the entire acceleration phase. The stick figures illustrate the body segment positions at the foot-strike and just before the toe-off from the 1st to 25th step. Numbers in the figure indicate the respective steps. The red figures overlapping the black figures are those immediately before the respective steps. The MP joint was used as a reference to adjust the horizontal positions of the stick figures of adjacent steps.

The times of 60-m sprints are given in Table 2. The standardised average sprinting motion at the foot-strike and just before the toe-off of the best trials of all 12 sprinters for 25 steps are shown in Fig. 2. As depicted in Fig. 2, sprinting motion changed step to step and the magnitude of the changes gradually became small toward the 25th step.

**Table 2.**
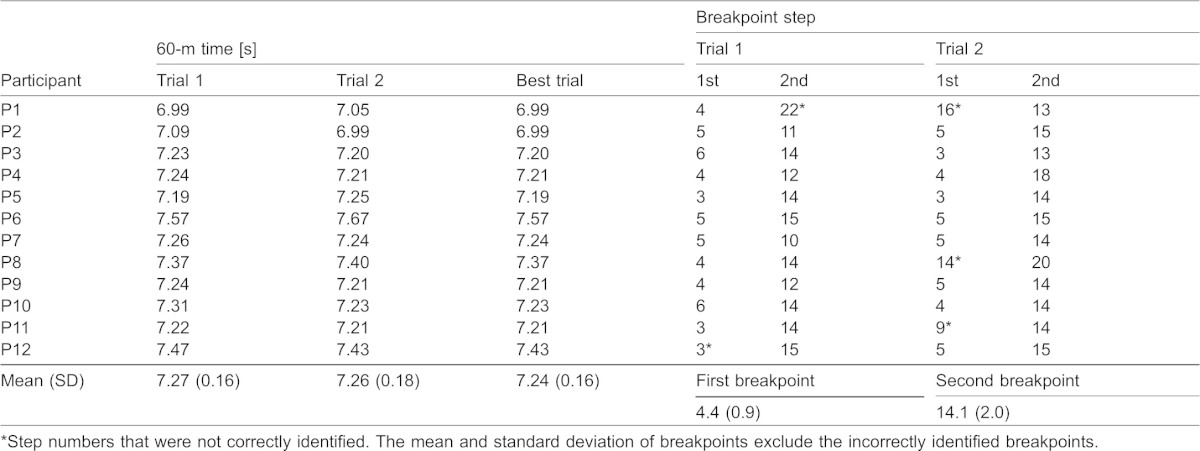
Times of 60-m sprints and the first and second breakpoints identified for all participants' trials

**Fig. 3.**
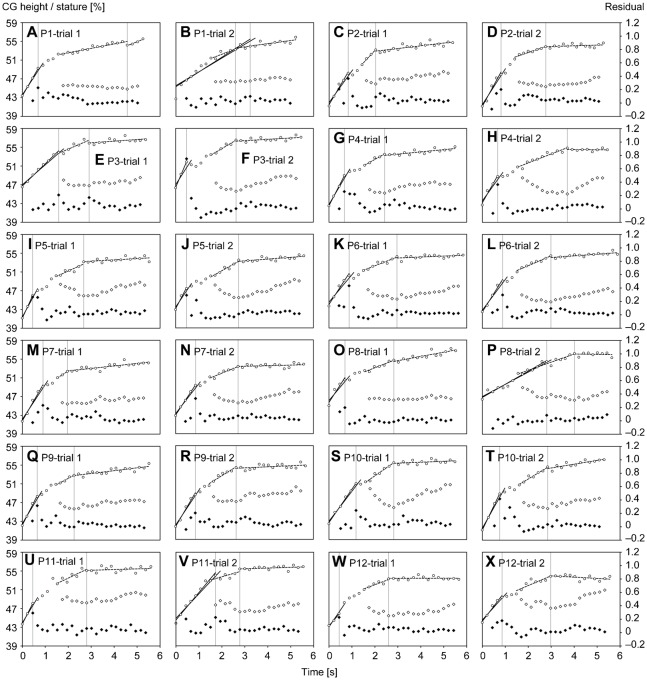
Changes in CGMH and the breakpoint detection in individuals' trials. The symbols have the same meaning as those in Fig. 1B.

Fig. 3 presents the breakpoint (transition step) detection for all individual trials. Using our proposed breakpoint detection procedures, the first breakpoints were correctly detected for 20 of 24 trials and ranged from the third to sixth step and the second breakpoints were correctly detected for 23 of 24 trials and ranged from the 10th to 20th step (Table 2). The step numbers (average ± standard deviation) of the correctly detected first and second breakpoints were 4.4±0.9 and 14.1±2.0, respectively.

### Changes in spatiotemporal and kinematic variables

**Fig. 4.**
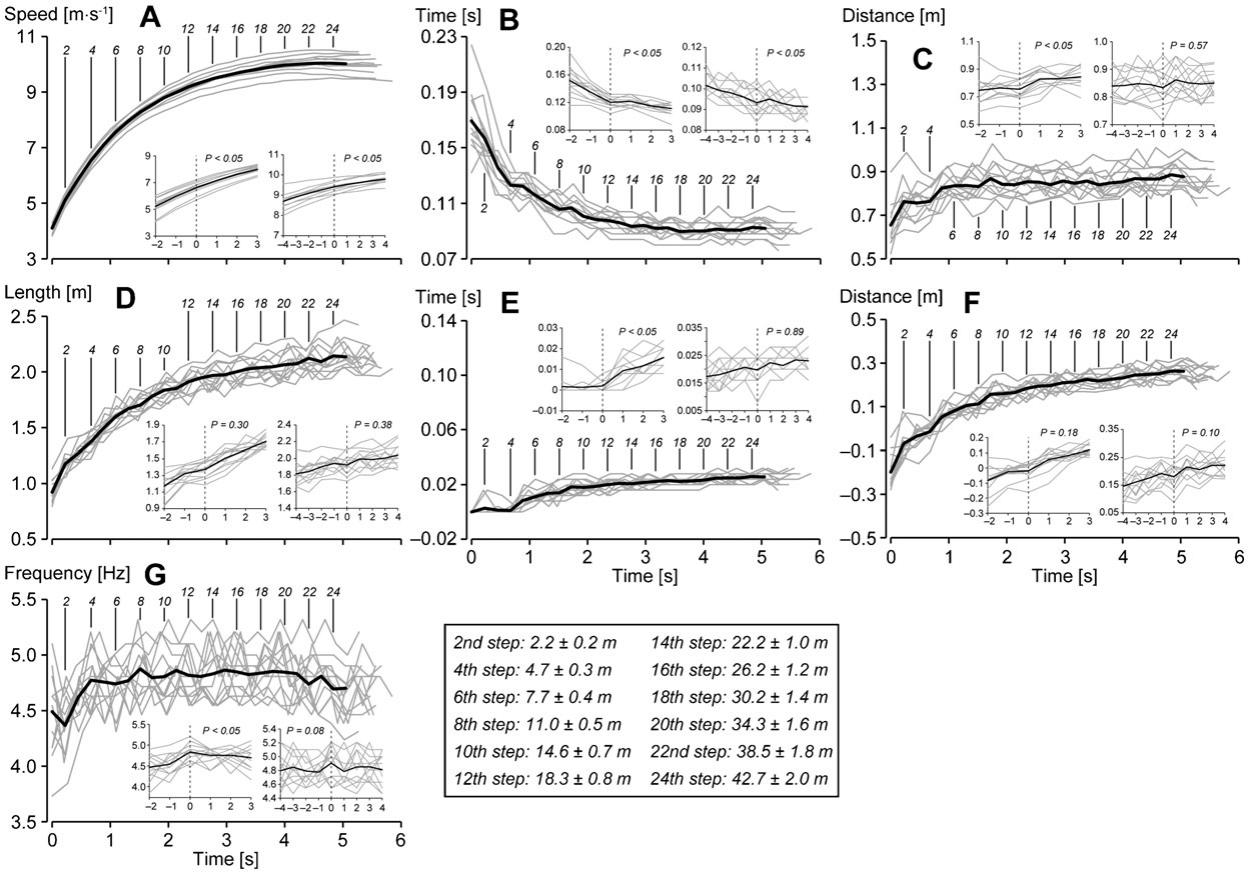
Changes in spatiotemporal variables during the entire acceleration phase of maximal sprinting. The sub-charts in the graphics show the changes in variables before (two steps before for the first breakpoint and four steps before for the second breakpoint) and after (three steps after for the first breakpoint and four steps after for the second breakpoint) the breakpoints. The averaged data are plotted against averaged times from the first foot-strike to the foot-strikes of the respective steps. Vertical solid lines with numbers indicate the positions of the respective number of steps for average values. The average distances from the start line to the respective steps (mid-position of a step) are shown at the centre on the bottom. (A) Running speed, (B) ST, (C) SD, (D) SL, (E) AST, (F) ASD, (G) SF. *P* values of the *t*-test of gradients of approximated values around the breakpoints are presented in respective sub-charts.

**Fig. 5.**
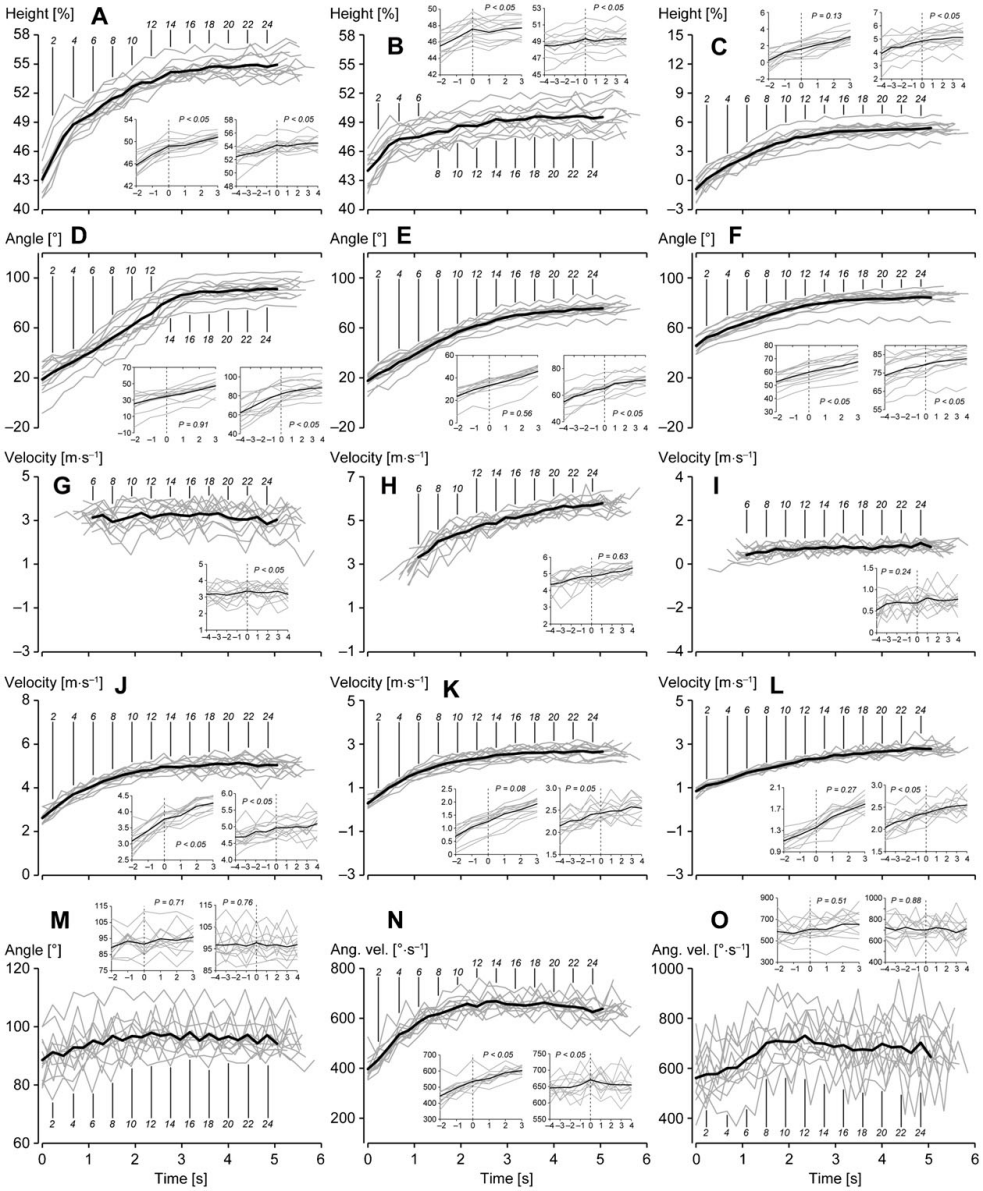
Changes in CG relative and kinematic variables during the entire acceleration phase of maximal sprinting. The symbols have the same meaning as those in Fig. 4. (A) CGMH, (B) hip-MH, (C) hip-CG-MH, (D) head angle, (E) upper-trunk angle, (F) lower-trunk angle, (G) thigh-VA, (H) shank-VA, (I) foot-VA, (J) thigh-VP, (K) shank-VP, (L) foot-VP, (M) hip angular displacement (maximum to minimum), (N) mean hip angular velocity during the support phase, (O) peak knee extension velocity during the support phase. *P* values of the *t*-test of gradients of approximated values around the breakpoints are presented in respective sub-charts. In the case of (G) thigh-VA, (H) shank-VA, and (I) foot-VA, variables during the early part of acceleration for some participants and the average are not shown because there was no anterior support phase during that part.

Figs 4 and 5 illustrate changes in spatiotemporal and kinematic measures during the entire acceleration phase. Means for each variable are the average values at respective steps for the best trials of 12 individuals. The sub-charts in each graphic of figures show the changes in variables before (two steps for the first breakpoint and four steps for the second breakpoint) and after (three steps for the first breakpoint and four steps for the second breakpoint) the breakpoints. In the case that the two breakpoints were correctly detected for two trials of an individual, the best trial was adopted to obtain the data prior to and after the breakpoints. For the participants P8, P11, and P12, the data of the first, first, and second trials, respectively, were used. Because both trials partially failed to detect the breakpoints, the data around the first and second breakpoints of the first and second trials, respectively, of participant P1 were used.

In accelerated sprinting, mean spatiotemporal variables of all individuals' best trials changed as follows. Running speed increased acutely during the initial phase and the magnitude of the increase became small (Fig. 4A), and changes in the SL had a profile similar to that of changes in running speed (Fig. 4D). The maximal values for the running speed and SL were, respectively, 10.04±0.29 m·s^−1^ at the 23rd step and 2.14±0.16 m at the 24th step. In contrast, SF increased until the fourth step and remained constant thereafter, although there was relatively large inter- and intra-individual variability (maximal value of 4.87±0.16 Hz at the eighth step) (Fig. 4G). ST decreased rapidly to the fourth step and levelled off to the fifth step, and then decreased again gradually until reaching a minimum value (0.090±0.006 seconds) at the 20th step (Fig. 4B). SD increased to the second step and levelled off to the fourth step, and then increased again to the fifth step and was roughly constant thereafter to a maximal value (0.89±0.06 m) at the 24th step (Fig. 4C). AST was almost zero until the fourth step and increased rapidly from the fifth step; the magnitude of the increase gradually became smaller and the value was maximal (0.026±0.004 seconds) at the 24th step (Fig. 4E). ASD was less than zero up to the fourth step, and increased beyond zero at the fifth step and then gradually increased until the 24th step (0.26±0.04 m) (Fig. 4F).

Around the first breakpoint, the gradients of changes in running speed, SF, ST, SD, and AST changed significantly. Around the second breakpoint, the gradients of changes in running speed and ST changed significantly.

As shown in Fig. 5A, CGMH increased rapidly until the fourth step and then more gradually until the 14th step, after which the CGMH remained approximately constant; the maximal value (54.9±1.5%) appeared at the 25th step. The mean height of the support hip relative to stature (hip-MH) increased acutely to the fourth step and gradually thereafter until the 23rd step (49.6±1.1%) (Fig. 5B). The mean height from the support hip to the CG in relation to stature (hip-CG-MH) increased until approximately the 15th step and remained approximately constant thereafter (maximal value of 5.4±0.7% at the 25th step) (Fig. 5C). The mean head angle during the support phase (hereafter simply referred to as the head angle) increased until around the 15th step, after which it remained approximately constant, reaching a maximal value at the 24th step (91.2±7.3°) (Fig. 5D). The mean upper-trunk angle during the support phase (hereafter simply referred to as the upper-trunk angle) increased to the 16th step and then more gradually; the maximal value was 75.7±5.6° at the 25th step (Fig. 5E). The mean lower-trunk angle during the support phase (hereafter simply referred to as the lower-trunk angle) increased gradually to the 16th step and then remained approximately constant; the maximal value was 84.7±7.4° at the 24th step (Fig. 5F). The mean contribution of the thigh to the running speed during the anterior support phase (thigh-VA) was roughly constant during the acceleration phase, although there was no anterior support phase during the early part of acceleration, and the maximal value was 3.35±0.52 m·s^−1^ at the 11th step (Fig. 5G). The mean contribution of the shank to the running speed during the anterior support phase (shank-VA) increased with the number of steps, reaching a maximal value at the 25th step (5.79±0.44 m·s^−1^) (Fig. 5H). The mean contribution of the foot to the running speed during the anterior support phase (foot-VA) was maintained during the acceleration phase; the maximal value was 0.97±0.27 m·s^−1^ at the 24th step (Fig. 5I). The mean contribution of the thigh to the running speed during the posterior support phase (thigh-VP) gradually increased until the 13th step and subsequently remained approximately constant; the maximal value was 5.14±0.42 m·s^−1^ at the 21st step (Fig. 5J). The mean contribution of the shank to the running speed during the posterior support phase (shank-VP) had a profile similar to that of the thigh during the first half of the acceleration phase and gradually levelled off; the maximal value was 2.68±0.33 m·s^−1^ at the 23rd step (Fig. 5K). The mean contribution of the foot to the running speed during the posterior support phase (foot-VP) increased at a slightly decreasing rate; the maximal value was 2.82±0.30 m·s^−1^ at the 23rd step (Fig. 5L). The hip angular displacement (maximum to minimum during the swing phase) gradually increased, reaching a maximal value (98.1±5.9°) at the 16th step, and slightly decreased thereafter (Fig. 5M). The mean hip angular velocity during the support phase increased and then gradually decreased; the maximal value was 668±31°·s^−1^ at the 14th step (Fig. 5N). The peak knee angular velocity during the support phase increased to the eighth step and then gradually decreased slightly; the maximal value was 730±73°·s^−1^ at the 12th step (Fig. 5O).

Around the first breakpoint, the gradients of changes in CGMH, hip-MH, lower-trunk angle, thigh-VP, and the mean hip angular velocity during the support phase significantly changed. Around the second breakpoint, the gradients of changes in CGMH, hip-MH, hip-CG-MH, head and upper- and lower-trunk angles, thigh-VA, thigh-VP, foot-VP, and the mean hip angular velocity during the support phase changed significantly.

## DISCUSSION

To our knowledge, this is the first time that the kinematics of sprinting has been measured through the entire acceleration phase. According to the results obtained in this study, the methods for breakpoint detection are validated and the characteristics of transition when sprinters cross the breakpoints are illustrated in the first part of the discussion. In the second part, different acceleration strategies in the sections determined by the breakpoints are briefly demonstrated according to the step-to-step changes in spatiotemporal and kinematic variables during the entire acceleration phase of maximal sprinting.

### Breakpoint detection and transitions and sections of accelerated sprinting

**Fig. 6.**
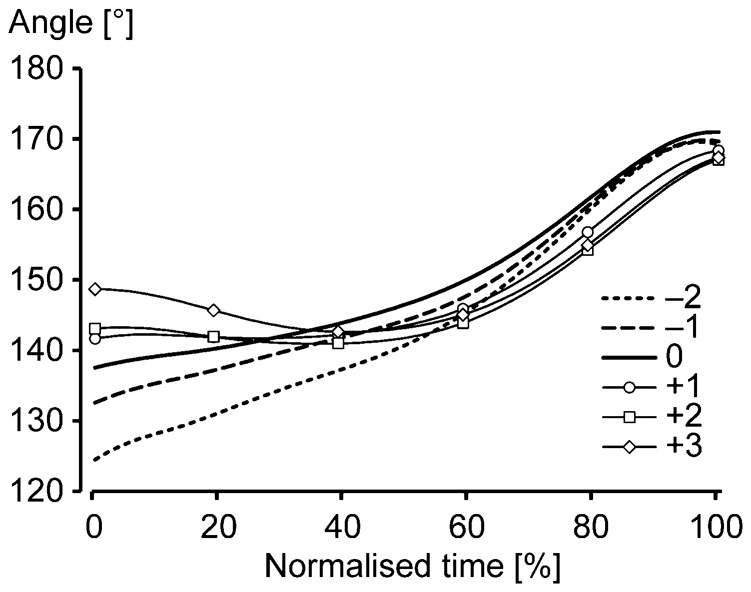
Changes in knee-joint angle before and after the first breakpoint. The line graphics indicate changes in joint angle during the support phase two steps before and three steps after the breakpoint step. Variables of the same steps that were adopted for sub-charts in Fig. 4 were used and, thus, the real step numbers of the used data among individuals were different. The values are average values for all participants.

Although the presented methods partially failed to detect the breakpoints, we found discriminable changes in the kinematics of sprinters, representing the transition relating to the crossing of our determined breakpoints, which enhances the validity of our methods. For the first transition, a typical alteration of spatiotemporal and kinematic measures can be seen in Fig. 4B,C,E,G and Fig. 5A,B,N. Fig. 6 shows the changes in a knee-joint angle during the support phase before and after the detected first breakpoint. The phenomenon before and after the first transition step was as follows. Sprinters accelerated with a rapid increase in SF and a rapid decrease in ST, contacting their foot on the ground behind the CG, a rapid increase in hip extension velocity, and no knee flexion during the support phase toward the first transition step, which indicates that the sprinters tried to accelerate step to step with pushing motion and increasing movement frequency until the transition step. In this process, the propulsive impulse that compensates the gravitational rotation force presumably suddenly could not be exerted (at the first transition step), and it might become necessary to change motion at the next step by contacting the foot far forward and starting to flex the knee during the support phase to keep balance. This adaptation probably resulted in a suspension of the decrease in ST (Fig. 4B), an abrupt increase in SD (Fig. 4C), and termination of the rapid increase in CG and hip heights (Fig. 5A,B). Whereas it is unclear how this adaptation of movement is controlled, the adaptation occurred during the swing phase according to the results of ASD. This change in ASD probably resulted in the knee flexion during the support phase, and the knee flexion during the support phase after the transition step presumably led to the production of larger forces at knee and ankle joints during the support phase, for a further increase in running speed, owing to stretch shortening cycle activity of extensor (plantar flexor) muscles at these joints (noting that knee flexion preceding extension during the support phase enhances the ankle plantar flexion) (Cavagna et al., 1971; Fukunaga et al., 1981). Moreover, although it is difficult to conclude the reason why the abrupt change happens, one possible reason is that the attractor of the human cyclic motion (Diedrich and Warren, 1995; Haken et al., 1985) keeps pulling in the sprinter's motion until the sprinter is unable to increase the movement frequency.

Figs 4 and 5 show that changes in sprinting motion, especially changes in head and trunk postures and CG height (Fig. 5A–F), became stable after sprinters crossed the second transition step, although the transition was gradual. Interestingly, the mean hip angular velocity during the support phase (Fig. 5N) decreased from the second transition step while running speed increased (hip angular displacement, as seen in Fig. 5M, also decreased from the second transition step, although there was no significant difference in gradient around the transition step). Additionally, thigh-VP levelled off from the second transition step (Fig. 5J). Along with the first transition, it is difficult to conclude the reason why these changes happen. However, it is obvious that the strategy of acceleration changed around the second transition step. Moreover, it is speculated that a stable upright trunk posture leads to increased muscle tension in front of the body, especially in the case of the iliopsoas muscle (Morini et al., 2008). Thus, sprinters seem to become able to swing the leg forward more quickly (Dorn et al., 2012) with a small range of motion possibly along with reduced hip extension velocity before the toe-off. Additionally, the increase in iliopsoas muscle tension during the early swing phase, tilting the pelvis forward, induces substantial hamstring stretch in the opposite limb (Chumanov et al., 2007). This hamstring stretch before the foot-strike has the potential to increase the shank backward velocity and propulsive impulse during the following support phase and thus increase the SL (Mero and Komi, 1987). Consequently, according to the concept that the stable trunk posture affects force production capability in lower extremities, it is plausible that the range (time or distance) of acceleration with inclined trunk posture is limited, and there is another acceleration strategy with stable upright trunk posture. Moreover, it seems that the stable upright posture leads the second transition during the entire acceleration of human maximal sprinting.

Previous studies have reported results comparable to the results of our study. Plamondon and Roy showed that the braking force and impulse abruptly increase from the fifth step (Plamondon and Roy, 1984). This abrupt increase in braking force can also be seen in the study by Morin et al., which showed the change in a typical GRF profile during accelerated sprinting on an instrumented self-driven treadmill (Morin et al., 2010). Moreover, Fukunaga et al. reported that the knee joint started to flex during the support phase from the fifth step in maximal accelerated sprinting (Fukunaga et al., 1981). These previous studies support our findings at the first transition. There is only one study that can be compared with our study regarding the variables at the second transition. Plamondon and Roy presented step-to-step changes in trunk angle during the acceleration phase and found that the angle became stable from the 14th step (Plamondon and Roy, 1984), which is in line with the results of our study. Additionally, the average step numbers of the two transitions are in accordance with the discriminating steps determined for different acceleration sections by Nagahara et al. (Nagahara et al., 2014), although the number of steps for the second transition slightly differs; the final section started at the 16th step in the previous study and the second transition step was at the 14th step in our study.

Our methods incorrectly detected the first and second breakpoints in 17% and 4% of trials, respectively. In the case of the first breakpoint, the steps determined for three trials (Fig. 3B,P,V) were beyond the expected range (up to the seventh step), although there visually appears to be another breakpoint at the second (Fig. 3B), second (Fig. 3P), and third steps (Fig. 3V). The remaining case (Fig. 3W) of the incorrect determination of the first breakpoint related to the peak value of the difference in mean absolute residuals indicating a change in gradient contrary to that expected; i.e. the breakpoint was determined where the change in CGMH became large rapidly, although we intended to detect the breakpoint where the change in CGMH becomes small. In the case of the incorrectly detected second breakpoint (Fig. 3A), the second approximation line was steeper than the first, which was not anticipated.

The findings of our study provide insight into the human locomotor system under an extreme condition; i.e. maximal accelerated sprinting. The fact that the breakpoints were determined within a relatively limited number of steps shows that the discriminable changes in kinematics—the transition—possibly result from some sort of constraints in the nature of the human locomotor system. Moreover, in regard to the first transition, which is likely inefficient, even though the participants in this study were very well trained sprinters, the phenomenon of the transition could be found. This indicates that the phenomenon may be inevitable. Although the kinematic aspects of transition in human maximal accelerated sprinting were revealed in our study, the kinematic changes are produced by kinetic changes and these are driven by muscle contraction. The speculated sources of the abrupt changes in kinematics around the transitions in this study should be confirmed by investigating the kinetics and an electromyogram.

### Spatiotemporal and kinematic characteristics of maximal accelerated sprinting

Three acceleration sections (the initial section, from the first step to the first breakpoint; the middle section, from the first breakpoint to the second breakpoint; the final section, from the second breakpoint to the step when the running speed became maximal), having different acceleration strategies, are determined according to our two detected breakpoints. In the initial section, sprinters contacted the foot on the ground behind the body and extended the hip and knee with increasing SL and SF to increase running speed (Fig. 4D,F,G, Fig. 5N, Fig. 6). Moreover, they elevated the CG rapidly with changes in the support leg (Fig. 5A,B) and trunk posture (the contribution of the leg to the elevation was relatively large) (Fig. 5C,E,F), and thigh-VP and shank-VP equivalently increased in the initial acceleration section (Fig. 5J,K). In the middle section, sprinters accelerated with increasing SL, and they contacted the foot on the ground in front of the body with flexion–extension movement of the knee during the support phase (Fig. 4E,F, Fig. 6). Additionally, the changes in head and trunk postures mainly raised the CG in the middle section (Fig. 5C–F). Sprinters increased shank-VA (Fig. 5H), and increases in thigh-VP, shank-VP, and foot-VP were almost the same in the middle acceleration section (Fig. 5J,K,L). Here, it would be better to note that the peak knee extension velocity during the support phase became stable after reaching the eighth step in the middle section as shown in Fig. 5O, which means that the active knee extension during the support phase terminated at that step. Thus, when focusing on this distinctive change in knee motion during the support phase, the middle acceleration section is possibly divided into two further parts. In the final section, the increase in running speed was still attributed to SL (Fig. 4D), and changes in the other spatiotemporal variables except for ASD were minimal (Fig. 4B,C,E,F). Furthermore, changes in CGMH and head and trunk angles were stable in the final section (Fig. 5A–F), while the range and velocity of the hip motion slightly decreased (Fig. 5M,N). To increase the running speed in the final acceleration section, only shank-VA and foot-VP increased (Fig. 5H,L). Altogether, the results obtained for segment contributions reveal that different segments are responsible for the accelerations in different sections in terms of contribution to the running speed; i.e. the thigh and shank in the initial section, the thigh, shank, and foot in the middle section, and the shank and foot in the final section. This supports the concept of different acceleration strategies in human maximal accelerated sprinting.

After the average running speed reached a maximum at the 23rd step, SL, shank-VA, and foot-VP continued to increase (Fig. 4D, Fig. 5H,L). Previous studies (Ae et al., 1992a; Gajer et al., 1999; Moravec et al., 1988) have demonstrated that the SL and flight time increase throughout a 100-m race, whereas the running speed increases and then decreases. Thus, continued increases in the variables observed in our study are in line with the results of the previous studies. These results demonstrate that the mechanism of running during the deceleration phase is different from that during the acceleration phase, even though the running speeds are the same.

As a limitation of the present study, although we verified the transitions during the entire acceleration phase of maximal sprinting for all participants (with partial failures of detections), all participants were well-trained sprinters. It would be of interest to investigate maximal sprinting during the entire acceleration phase for non-trained adults or children to confirm the transitions to be a result of constraints of the human locomotor system.

### Conclusion

This study documented the step-to-step changes in kinematics of human maximal sprinting during the entire acceleration phase. According to the changes in CG height, two breakpoints during the entire acceleration phase were detected. Discriminable kinematic changes were found when the sprinters crossed the detected first transition—the foot starting to contact the ground in front of the CG, the knee-joint starting to flex during the support phase and the termination of the increase in step frequency with suspension of the decrease in support time—and second transition—the termination of changes in body postures and the start of a slight decrease in the intensity of hip-joint movements. Consequently, we conclude that there are probably two transitions during human maximal accelerated sprinting. In the different acceleration sections delimited by transition steps, different acceleration strategies, especially changes in the contributions of lower-extremity segments (i.e. thigh and shank for the initial section, thigh, shank, and foot for the middle section, and shank and foot for the final section), are employed to increase running speed.

## References

[b1] Ae M. Ito A. Suzuki M. (1992a). The men's 100 metres. New Studies in Athletics 7, 47–52.

[b2] Ae M. Tang H. P. Yokoi T. (1992b). Estimation of inertia properties of the body segment in Japanese athletes. Biomechanisms 11, 23–33.

[b3] Ae M. Muraki Y. Koyama H. Fujii N. (2007). A biomechanical method to establish a standard motion and identify critical motion by motion variability: with examples of high jump and sprint running. Bulletin of Institute of Health and Sport Science 30, 5–12.

[b4] Bezodis I. N. Kerwin D. G. Salo A. I. (2008). Lower-limb mechanics during the support phase of maximum-velocity sprint running. Med. Sci. Sports Exerc. 40, 707–715 10.1249/MSS.0b013e318162d162 18317373

[b5] Bezodis N. E. Salo A. I. Trewartha G. (2014). Lower limb joint kinetics during the first stance phase in athletics sprinting: three elite athlete case studies. J. Sports Sci. 32, 738–746 10.1080/02640414.2013.849000 24359568

[b6] Bosch F. Klomp R.(2005). Running techniques. Running: Biomechanics and Exercise Physiology Applied in Practice Bosch F Klomp R, (translated by D. W. Boer-Stallman), 119–188 Philadelphia, PA: Elsevier, (original work published in 2001).

[b7] Bret C. Rahmani A. Dufour A. B. Messonnier L. Lacour J. R. (2002). Leg strength and stiffness as ability factors in 100 m sprint running. J. Sports Med. Phys. Fitness 42, 274–281. 12094115

[b8] Bruggemann G. P. Glad B. (1990). Time analysis of the sprint events. Scientific research project at the Games of the XXIVth Olympiad – Seoul 1988: final report. New Studies in Athletics 1, 11–89.

[b9] Cavagna G. A. Komarek L. Mazzoleni S. (1971). The mechanics of sprint running. J. Physiol. 217, 709–721. 5098087 10.1113/jphysiol.1971.sp009595 PMC1331572

[b10] Celik H. Piazza S. J. (2013). Simulation of aperiodic bipedal sprinting. J. Biomech. Eng. 135, 081008 10.1115/1.4024577 23722442

[b11] Chapman A. E. Caldwell G. E. (1983). Kinetic limitations of maximal sprinting speed. J. Biomech. 16, 79–83 10.1016/0021-9290(83)90048-9 6833312

[b12] Charalambous L. Irwin G. Bezodis I. N. Kerwin D. (2012). Lower limb joint kinetics and ankle joint stiffness in the sprint start push-off. J. Sports Sci. 30, 1–9 10.1080/02640414.2011.616948 22098532

[b13] Chumanov E. S. Heiderscheit B. C. Thelen D. G. (2007). The effect of speed and influence of individual muscles on hamstring mechanics during the swing phase of sprinting. J. Biomech. 40, 3555–3562 10.1016/j.jbiomech.2007.05.026 17659291

[b14] Debaere S. Jonkers I. Delecluse C. (2013a). The contribution of step characteristics to sprint running performance in high-level male and female athletes. J. Strength Cond. Res. 27, 116–124 10.1519/JSC.0b013e31825183ef 22395270

[b15] Debaere S. Delecluse C. Aerenhouts D. Hagman F. Jonkers I. (2013b). From block clearance to sprint running: characteristics underlying an effective transition. J. Sports Sci. 31, 137–149 10.1080/02640414.2012.722225 22974278

[b16] Delecluse C. (1997). Influence of strength training on sprint running performance. Current findings and implications for training. Sports Med. 24, 147–156 10.2165/00007256-199724030-00001 9327528

[b17] Diedrich F. J. Warren W. H. Jr (1995). Why change gaits? Dynamics of the walk-run transition. J. Exp. Psychol. Hum. Percept. Perform. 21, 183–202 10.1037/0096-1523.21.1.183 7707029

[b18] Dillman C. J. (1975). Kinematic analyses of running. Exerc. Sport Sci. Rev. 3, 193–218 10.1249/00003677-197500030-00010 1175666

[b19] Doolittle D. Tellez T. (1984). Sprinting – from start to finish. Track and Field Quarterly Review 84, 5–8.

[b20] Dorn T. W. Schache A. G. Pandy M. G. (2012). Muscular strategy shift in human running: dependence of running speed on hip and ankle muscle performance. J. Exp. Biol. 215, 1944–1956 10.1242/jeb.064527 22573774

[b21] Eames M. H. A. Cosgrove A. Baker R. (1999). Comparing methods of estimating the total body centre of mass in three-dimensions in normal and pathological gaits. Hum. Mov. Sci. 18, 637–646 10.1016/S0167-9457(99)00022-6

[b22] Fukunaga T. Matsuo A. Ichikawa M. (1981). Mechanical energy output and joint movements in sprint running. Ergonomics 24, 765–772 10.1080/00140138108924898 7318811

[b23] Gajer B. Thépaut-Mathieu C. Lehénaff D. (1999). Evolution of stride and amplitude during course of the 100m event in athletics. New Studies in Athletics 14, 43–50.

[b24] Haken H. Kelso J. A. Bunz H. (1985). A theoretical model of phase transitions in human hand movements. Biol. Cybern. 51, 347–356 10.1007/BF00336922 3978150

[b25] Hirvonen J. Rehunen S. Rusko H. Härkönen M. (1987). Breakdown of high-energy phosphate compounds and lactate accumulation during short supramaximal exercise. Eur. J. Appl. Physiol. Occup. Physiol. 56, 253–259 10.1007/BF00690889 3569234

[b26] Hreljac A. Marshall R. N. (2000). Algorithms to determine event timing during normal walking using kinematic data. J. Biomech. 33, 783–786 10.1016/S0021-9290(00)00014-2 10808002

[b27] Hunter J. P. Marshall R. N. McNair P. J. (2004). Interaction of step length and step rate during sprint running. Med. Sci. Sports Exerc. 36, 261–271 10.1249/01.MSS.0000113664.15777.53 14767249

[b28] Hunter J. P. Marshall R. N. McNair P. J. (2005). Relationships between ground reaction force impulse and kinematics of sprint-running acceleration. J. Appl. Biomech. 21, 31–43. 16131703 10.1123/jab.21.1.31

[b29] Jacobs R. van Ingen Schenau G. J. (1992). Intermuscular coordination in a sprint push-off. J. Biomech. 25, 953–965 10.1016/0021-9290(92)90031-U 1517272

[b30] Jensen R. K. (1978). Estimation of the biomechanical properties of three body types using a photogrammetric method. J. Biomech. 11, 349–358 10.1016/0021-9290(78)90069-6 711783

[b31] Johnson M. D. Buckley J. G. (2001). Muscle power patterns in the mid-acceleration phase of sprinting. J. Sports Sci. 19, 263–272 10.1080/026404101750158330 11311024

[b32] Jones R. H. Molitoris B. A. (1984). A statistical method for determining the breakpoint of two lines. Anal. Biochem. 141, 287–290 10.1016/0003-2697(84)90458-5 6496934

[b33] Kugler F. Janshen L. (2010). Body position determines propulsive forces in accelerated running. J. Biomech. 43, 343–348 10.1016/j.jbiomech.2009.07.041 19863962

[b34] Kunz H. Kaufmann D. A. (1981). Biomechanical analysis of sprinting: decathletes versus champions. Br. J. Sports Med. 15, 177–181 10.1136/bjsm.15.3.177 7272662 PMC1858761

[b35] Maćkala K. (2007). Optimisation of performance through kinematic analysis of the different phases of the 100 metres. New Studies in Athletics 22, 7–16.

[b36] Mann R. Herman J. (1985). Kinematic analysis of Olympic sprint performance: men's 200 meters. J. Appl. Biomech. 1, 151–162.

[b37] Margaria R. Aghemo P. Rovelli E. (1966). Measurement of muscular power (anaerobic) in man. J. Appl. Physiol. 21, 1662–1664. 5923240 10.1152/jappl.1966.21.5.1662

[b38] Mero A. Komi P. V. (1987). Electromyographic activity in sprinting at speeds ranging from sub-maximal to supra-maximal. Med. Sci. Sports Exerc. 19, 266–274 10.1249/00005768-198706000-00014 3600241

[b39] Mero A. Komi P. V. Gregor R. J. (1992). Biomechanics of sprint running. A review. Sports Med. 13, 376–392 10.2165/00007256-199213060-00002 1615256

[b40] Moravec P. Ruzicka J. Susanka P. Dostal E. Kodejs M. Nosek M. (1988). The 1987 International Athletic Foundation/IAAF Scientific Project Report: Time analysis of the 100 metre events at the II World Championships in Athletics. New Studies in Athletics 3, 61–96.

[b41] Morin J. B. Samozino P. Bonnefoy R. Edouard P. Belli A. (2010). Direct measurement of power during one single sprint on treadmill. J. Biomech. 43, 1970–1975 10.1016/j.jbiomech.2010.03.012 20541762

[b42] Morin J. B. Bourdin M. Edouard P. Peyrot N. Samozino P. Lacour J. R. (2012). Mechanical determinants of 100-m sprint running performance. Eur. J. Appl. Physiol. 112, 3921–3930 10.1007/s00421-012-2379-8 22422028

[b43] Morini S. Ciccarelli A. Cerulli C. Giombini A. Di Cesare A. Ripani M. (2008). Functional anatomy of trunk flexion-extension in isokinetic exercise: muscle activity in standing and seated positions. J. Sports Med. Phys. Fitness 48, 17–23. 18212705

[b44] Nagahara R. Zushi K. (2013). Determination of foot strike and toe-off event timing during maximal sprint using kinematic data. International Journal of Sport and Health Science 11, 96–100 10.5432/ijshs.201318

[b45] Nagahara R. Naito H. Morin J. B. Zushi K. (2014). Association of acceleration with spatiotemporal variables in maximal sprinting. Int. J. Sports Med (Epub ahead of print). 10.1055/s-0033-1363252 24577864

[b46] Neder J. A. Stein R. (2006). A simplified strategy for the estimation of the exercise ventilatory thresholds. Med. Sci. Sports Exerc. 38, 1007–1013 10.1249/01.mss.0000218141.90442.6c 16672856

[b47] Novacheck T. F. (1998). The biomechanics of running. Gait Posture 7, 77–95 10.1016/S0966-6362(97)00038-6 10200378

[b48] Nummela A. Rusko H. Mero A. (1994). EMG activities and ground reaction forces during fatigued and nonfatigued sprinting. Med. Sci. Sports Exerc. 26, 605–609 10.1249/00005768-199405000-00013 8007809

[b49] Plamondon A. Roy B. (1984). [Kinematics and kinetics of sprint acceleration]. Can. J. Appl. Sport Sci. 9, 42–52. 6705128

[b50] Schneider D. A. Phillips S. E. Stoffolano S. (1993). The simplified V-slope method of detecting the gas exchange threshold. Med. Sci. Sports Exerc. 25, 1180–1184 10.1249/00005768-199310000-00015 8231764

[b51] Segers V. Aerts P. Lenoir M. De Clercq D. (2007). Dynamics of the body centre of mass during actual acceleration across transition speed. J. Exp. Biol. 210, 578–585 10.1242/jeb.02693 17267643

[b52] Slawinski J. Bonnefoy A. Levêque J. M. Ontanon G. Riquet A. Dumas R. Chèze L. (2010). Kinematic and kinetic comparisons of elite and well-trained sprinters during sprint start. J. Strength Cond. Res. 24, 896–905 10.1519/JSC.0b013e3181ad3448 19935105

[b53] Slawinski J. Dumas R. Cheze L. Ontanon G. Miller C. Mazure-Bonnefoy A. (2013). Effect of postural changes on 3D joint angular velocity during starting block phase. J. Sports Sci. 31, 256–263 10.1080/02640414.2012.729076 23062070

[b54] van Ingen Schenau G. J. de Koning J. J. de Groot G. (1994). Optimisation of sprinting performance in running, cycling and speed skating. Sports Med. 17, 259–275 10.2165/00007256-199417040-00006 8009139

[b55] Volkov N. I. Lapin V. I. (1979). Analysis of the velocity curve in sprint running. Med. Sci. Sports 11, 332–337. 530024

[b56] Wells R. P. Winter D. A. (1980). Assessment of signal and noise in the kinematics of normal, pathological, and sporting gaits. Human Locomotion I. Proceedings of the Special Conference of the Canadian Society of Biomechanics 92–93 London, ON: Canadian Society of Biomechanics.

[b57] Weyand P. G. Sternlight D. B. Bellizzi M. J. Wright S. (2000). Faster top running speeds are achieved with greater ground forces not more rapid leg movements. J. Appl. Physiol. 89, 1991–1999. 11053354 10.1152/jappl.2000.89.5.1991

[b58] Young W. Benton D. Duthie G. Pryor J. (2001). Resistance training for short sprints and maximum-speed sprints. Strength and Conditioning Journal 23, 7–13 10.1519/00126548-200104000-00001

